# The effect of social support on home isolation anxiety and depression among college students in the post-pandemic era: the mediating effect of perceived loss of control and the moderating role of family socioeconomic status

**DOI:** 10.3389/fpubh.2024.1288848

**Published:** 2024-02-09

**Authors:** Hui Shi

**Affiliations:** School of Media and Communication, Shanghai Jiao Tong University, Shanghai, China

**Keywords:** social support, post-pandemic era, college students, depression and anxiety, perceived loss of control, family socioeconomic status

## Abstract

**Background:**

There is an escalating concern about the rising levels of anxiety and depression among college students, especially during the post-pandemic era. A thorough examination of the various dimensions of social support and their impact on these negative emotions in college students is imperative.

**Aim:**

This study aimed to determine if a perceived loss of control mediates the relationship between social support and levels of anxiety and depression among college students during the post-pandemic era. Additionally, it examined whether family socioeconomic status moderates this mediated relationship.

**Methods:**

We administered an online cross-sectional survey in China, securing responses from 502 participants. The sample comprised home-isolated college students impacted by COVID-19. Established scales were employed to assess social support, anxiety, depression, perceived loss of control, and family socioeconomic status. Analytical techniques included descriptive statistics, correlation analysis, and a bootstrap method to investigate mediating and moderating effects.

**Results:**

Social support was found to negatively affect anxiety and depression in college students, with perceived loss of control partially mediating this relationship. In addition, family socio-economic status was shown to moderate this moderating process. Furthermore, family socioeconomic status influenced this mediation, with higher socioeconomic families exhibiting a stronger moderating effect on perceived loss of control across different dimensions of social support.

**Conclusion:**

This study may help to develop strategies to mitigate the impact of anxiety and depression in the lives and studies of university students during unexpected public health crises, and to promote better mental health among college students.

## 1 Introduction

From the onset of the swift proliferation of the COVID-19 virus, it has aroused a broad spectrum of global attentiveness. Following suit, individuals have embarked upon a phase of production and living termed the “Post-Pandemic Era,” whose profound ramifications reverberate globally (1–4). The Post-Pandemic Era does not signify a complete eradication or total recuperation from the pandemic; rather, it denotes a period wherein the pandemic may still manifest in cyclical and scalable outbreaks, enduring over an extended duration, and perpetually impacting various domains (5–7). In March 2022, influenced by the Omicron variant of COVID-19, numerous regions in China witnessed a dramatic escalation in infection numbers (8, 9). In response to the outbreak, several local governments intensified their efforts in implementing “collective isolation” and “home isolation” measures (10, 11). Especially, the “citywide static management” measures adopted by Shanghai have elicited widespread public concern and attention (12). Although such measures exhibit significant efficacy in curtailing the dissemination of the virus (13, 14), the accompanying requisition for a vast populace to adhere to home isolation, the strain on medical resources, the scarcity of daily necessities, and the precipitous ascent in prices, have all contributed to the escalating anxiety amongst the citizenry (15–17). Some studies posit that the inadequacy of medical resources and the rapid augmentation of infection numbers are the principal catalysts inciting adverse repercussions on public mental health (18). Yet, there are perspectives that highlight the prolongation of home isolation and the frequent use of social media as pivotal factors also leading to the intensification of negative sentiments (19–21).

It is noteworthy that in the Post-Pandemic Era, compared to the resilience and positive outlook displayed during the initial phase of the pandemic, the populace tends to exhibit a more psychologically fragile demeanor during periods of home isolation (22, 23). The isolation policies and scarcity of resources are perceived as primary catalysts triggering and exacerbating psychological issues (24). Post-traumatic stress disorders are commonly manifested in individuals after encountering exceptional threats or calamities (25, 26). Anxiety and depression constitute two significant facets of this manifestation, often coexisting within the same individual either concurrently or at different junctures (27–29). Some studies assert that they are distinctly different, independent entities (30, 31). However, other studies have discerned that they represent overlapping syndromes, manifesting at different points on a phenomenological or temporal continuum, sharing common characteristics with essentially analogous clinical presentations (32–35). Psychological experts have discovered that post public health crisis, the comorbidity rate of anxiety and depression escalates to 60–70% (36). A substantial portion of COVID-19 patients exhibit symptoms indicative of a mixed anxiety-depression condition (37). Research indicates that during the spread of the pandemic in the first half of 2022 in China, the rapid proliferation and persistence of the COVID-19 virus posed a series of psychological challenges to the public, particularly in the comorbid manifestation of anxiety and depression. The clinical features generally encompass pessimism, sorrow, fear, concern, along with a loss of interest and vitality (38).

These emotions not only impact individuals’ psychological well-being, but may also jeopardize physical health through interference with immune and endocrine functions (39–42). College students, representing a vulnerable faction amidst this pandemic, have manifested as a high-risk populace for anxiety and depression (43). The outbreak’s emergence chanced upon the season of Chinese students returning to academia, where the abrupt instigation of isolation policies left numerous students marooned within their homes, hotels, or proximate to their institutions, in anticipation of quarantine cessation (44, 45). The pandemic’s instability within the Post-Pandemic Era further incites emotional fluctuations among university students (46, 47). Moreover, the decline in psychological well-being levies a hefty toll on society, families, and individuals (48, 49). Therefore, devising effective psychological intervention measures to address the mental challenges brought forth by home isolation during this era is of paramount importance. These initiatives aim to confront the academic and life adversities encountered by students both online and offline during home isolation, bolstering their psychological resilience, aiding them in overcoming the impacts of anxiety and depression, and rekindling their zeal and motivation towards academia and life.

Social support embodies the composite resources an individual garners within a social milieu, unveiling the intimate interaction between the individual and society (50, 51). Such support not only facilitates the redistribution of resources but also furnishes material and psychological sustenance for individuals amidst adversities, aiding in the mitigation of negative emotional onslaughts (52, 53). Studies delineate that amidst the Epidemic prevention and control, social support can significantly diminish residents’ anxiety (54). Elevated social support signifies heightened societal concern towards individual health, thereby attenuating negative emotional experiences (55). Further discoveries elucidate that the linkage between social support and psychological well-being is modulated by cognitive and expressive modalities, where proactively leveraging social support assists individuals in adopting more apt emotional regulation strategies. Compared to those with lower perceived social support, individuals with higher perceived support witnessed a 63% reduction in depression risk (56). This support predominantly emanates from family, friends, and other supportive connections, yet extant research chiefly centers on the psychological health impacts of family and friends on individuals in the post-pandemic epoch (57–59). Some studies suggest that the role of social groups and communities in alleviating the effects on individuals during pandemics remains contentious (60–62). Research regarding the assistance of communities and other similar entities is still notably lacking. Amid the advent of public health emergencies, other social supports can furnish individuals with critical resources like medical aid and materials, or facilitate resource interchange, factors that are quintessential for individuals’ productivity and livelihood during home isolation. Therefore, during the post-pandemic phase, a better comprehension of perceived social support across different ecological dimensions for individuals undergoing home isolation is of pivotal importance. In the post-pandemic epoch, China has instituted a networked management strategy rooted in community engagement, wherein streets and communal spheres have become the bedrock of residents’ daily endeavors (63). Amid abrupt epidemic onslaughts, students find themselves compelled into a state of dispersed isolation, rendering the community grid-based governance a pivotal adjunct of support during such junctures. Therefore, drawing from the aforementioned studies, we postulate that during the span of home isolation, the perceived social support among Chinese university students exhibits a negative correlation with their anxiety and depressive symptomatology.

When individuals harbor the conviction that they possess the capability to steer the outcomes of events, envisaging effective methodologies and indeed possessing such methodologies, they experience a sense of control (64). Conversely, sentiments such as hopelessness, helplessness, and diminished self-efficacy manifest a perceived loss of control, serving as potent conduits to depression (65, 66). Certain inquiries posit that unpredictable adversities could engender a perceived loss of control in individuals, subsequently precipitating a decline in the perceived meaningfulness of existence (67). Extant research delineates that perceived loss of control mediates the nexus between uncontrollable stressors and substance abuse (68). A prolonged engagement with a perceived loss of control could propel individuals into a chasm of hopelessness, ensnaring them in a tempest of negative emotions, and rendering them incapable of envisioning plans for their future existence (69, 70). Though some studies suggest that social support may ameliorate the anxiety, depression, and insomnia experienced by university students during the pandemic by bolstering individual self-control capacities (56, 71), unforeseen instances of home isolation, shortages in essential commodities and medical resources, coupled with a downturn in familial economic conditions, may plunge individuals into a profound sense of perceived loss of control (72, 73). Research has unveiled that amidst the COVID-19 era, university students are grappling with a salient psychological quandary of losing normalcy, with loss of control and avoidance emerging as primary determinants impacting mental well-being (74). Among them, medical students during the COVID-19 tenure, encounter difficulties in attaining relaxation and a sense of control, necessitating psychological interventions to ameliorate their mental tribulations (75). Further studies have discerned that those students with pre-existing health conditions may confront a dearth of medical resources during isolation, rendering their survival milieu increasingly stringent, which in turn may precipitate a further decline in their sense of control, potentially exacerbating their health statuses (76–78). Based on the aforementioned perspectives, we posit the first hypothesis in this study.

*H1*: Social support can reduce anxiety and depression among college students in long-term home isolation.

Moreover, social support can empower individuals to enhance their sense of control over external circumstances, leveraging the aid of others to alleviate their own perceived loss of control. Therefore, we propose a second hypothesis.

*H2*: Social support alleviates college students’ anxiety and depression by reducing their perceived loss of control.

Family socioeconomic status (SES) comprehensively reflects the status of a family’s core members in terms of economic resources, social hierarchy, and societal prestige (79). This status is not only a reflection of social stratification but also plays a crucial role throughout an individual’s life, profoundly impacting their growth and development (80, 81). Social support theory suggests that the impact of social support on individual psychological health can lead to different outcomes in specific contexts (82). The accessibility of social support is not entirely influenced by the social environment. Some studies have highlighted that individual differences play a role in the extent of social support received, which can have varying effects in different situations (83). For instance, within the same context of social support, groups with a lower socioeconomic status have been observed to have higher incidences of certain diseases and disabilities compared to those with higher socioeconomic statuses (84).

Theoretical research on family socioeconomic status demonstrates a significant correlation between varying social statuses and psychological health issues (85). Of particular concern is the intimate link between low socioeconomic status and psychological health problems, notably marked by an increased risk of loss of psychological control (86). Studies indicate that in families with lower socioeconomic status, the probability of developing psychological disorders such as anxiety and depression is substantially heightened (87, 88); on the other hand, a higher socioeconomic status might play a role in alleviating or ameliorating these issues of mental health issues (89).

Research during periods of family isolation indicates uneven distribution of resources across communities, leading to heightened tension and anxiety among residents. For instance, upscale neighborhoods in city centers may have access to special supply menus, a privilege not extended to other regular communities (90). For college students, despite their independence, they still rely on their families’ financial support (91). The economic condition of the family often determines the quantity and quality of social resources accessible to these students. Especially in the post-pandemic era of family isolation, family socioeconomic status emerges as a key influencing factor (92). Students from economically disadvantaged backgrounds might feel more isolated due to a lack of social resources, exacerbating feelings of anxiety and depression (93, 94). This highlights the importance of social resources during crises and the impact of family background on mental health. Based on these observations, we propose the following hypotheses.

*H3*: Family socioeconomic status plays a moderating role in the effect of social support on college students’ perceived loss of control, i.e., higher family socioeconomic status moderates college students’ perceived loss of control more.

*H4*: Family socioeconomic status plays a moderating role in the effect of social support on college students’ anxiety and depression, i.e., higher family socioeconomic status has a greater moderating role in the effect of social support on college students’ anxiety and depression.

**Figure 1:**
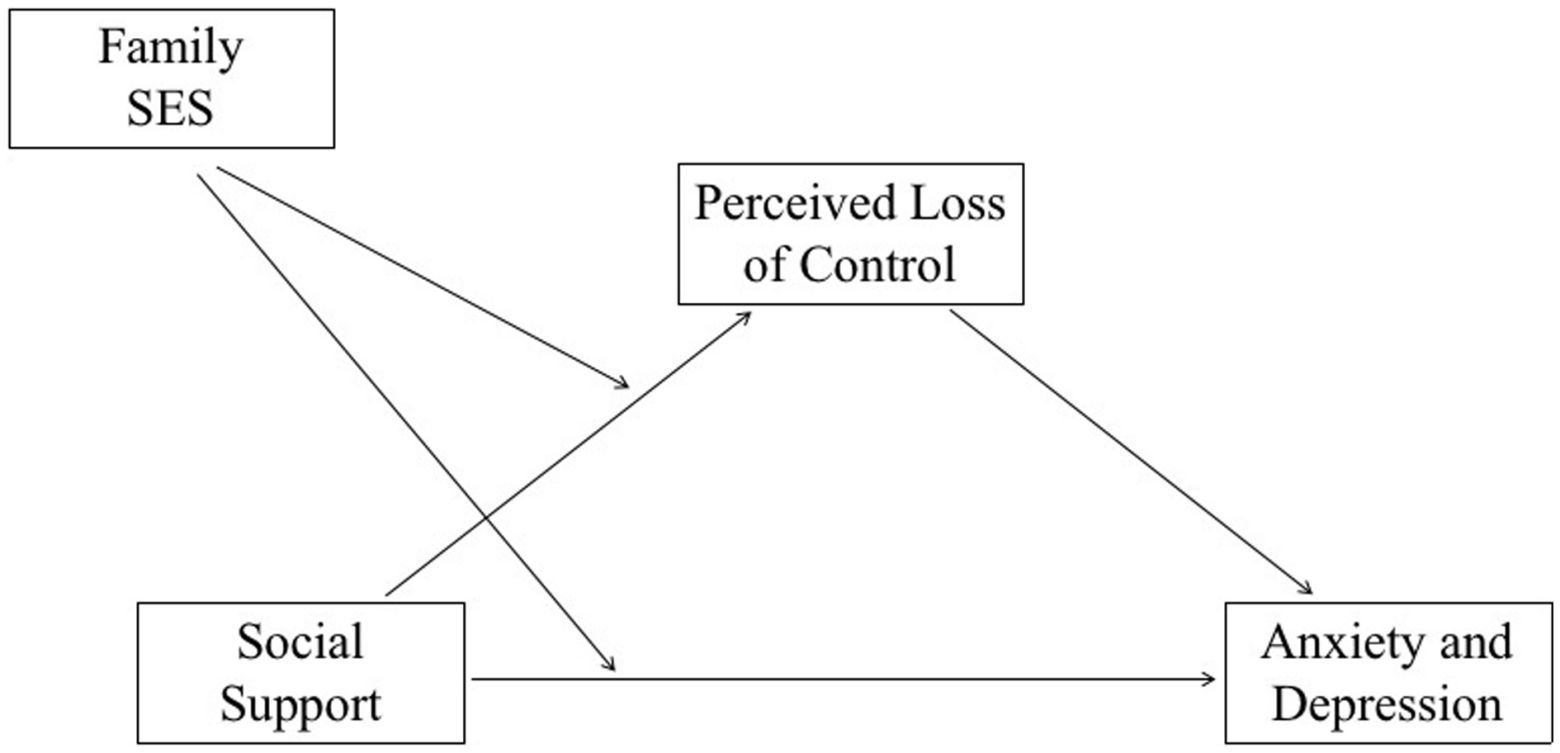
Research hypothesis model diagram.

The research hypothesis model diagram is shown in Figure 1.

## 2 Methods

### 2.1 Process and participants of the survey

#### 2.1.1 Design and procedure

This study employed a cross-sectional design, utilizing the online survey platform “Wenjuanxing”,^1^ to analyze the current status and relationships among the variables. Our survey targets were university students aged 18 and above residing in China. Inclusion criteria encompassed university students aged 18 and above, residing in China in areas under residential isolation due to the impact of COVID-19, and expressing willingness to participate in the online survey. Additionally, exclusion criteria were stipulated. The data collection spanned from April 1 to May 1, 2022, coinciding with the outbreak of the COVID-19 Omicron variant in China. During this period, the Chinese government implemented residential isolation measures in multiple areas to safeguard public health. Before participating in the survey, we provided the respondents with an informed consent form, requesting them to fill it out. Ultimately, we successfully collected 502 valid questionnaires. The survey tools included four scales, and we also collected basic demographic information from the participants.

#### 2.1.2 Sample characteristics

Of the 502 respondents included in this study, 259 were male and 243 were female. The age range was 18–32 for 470 (94%), and 32 (6%) were over 32 years old. Of these, 205 were undergraduate students (including high school and middle school), 135 were master’s students, and 62 were doctoral students. The isolation showed that isolation with family was 252, isolation with friends (including classmates, roommates, etc.) was 173, and isolation alone was 77. Isolation with family (50%) was the highest and isolation alone (15%) was the lowest.

### 2.2 Measurement

Dambi’s revised Perceived Social Support Scale (PSSS) was used (95), which has a total of 12 entries and is scored on a 5-point Likert scale (1 = completely disagree, to 5 = completely agree). The dimensions are categorized into three dimensions: family support, friend support, and other support. The scale is a cumulative score, with higher scores indicating a higher level of social support for the subjects. Taking into account the specific characteristics of university students in home isolation during the epidemic, we added, for example, “The neighborhood committee/street/school gave me enough support during the isolation period” as a question item for other support. In this study, the Cronbach’s α coefficient was 0.956.

The perceived loss of control was measured using a scale developed by Wen (96), the Chinese version of which has been shown to be reliable (97). The scale consists of 17 items, such as “I feel powerless to do anything.” A 5-point Likert scale was used (1 = not at all to 5 = fully), and the Cronbach’s coefficient in this study was 0.968.

Anxiety and depression were measured using a scale developed by Augustine (DASS-21) (98) with 14 items such as “I find it difficult to calm down.” A 5-point Likert scale was used (1 = not at all to 5 = fully). The total scale score was the sum of the scores for each question. The higher the score, the more intense the subject’s anxiety. In this study, the Cronbach’s α coefficient was 0.970.

Family socioeconomic status (SES) was measured using a scale developed by Ren Chunrong (99), the Chinese version of which has been shown to be reliable (100). The scale includes parents’ occupation, income, and education level, of which the income subscale is divided into 5 levels, with the higher the level, the higher the score (1 being the lowest and 5 being the highest); parents’ education level is divided into 5 categories, with the value of 1–5 points assigned sequentially; and occupation is divided into 5 levels, with the value of 1–5 points assigned sequentially. In the comprehensive method, the factor analysis method is used to calculate the socioeconomic status index of the individual’s family, and the formula is as follows:


$$FamilySES=\frac{\beta_{1}\times {Ζ}_{educationlevel}+\beta_{2}\times {Ζ}_{occupation}+\beta_{3}\times {Ζ}_{familyincome}}{Firsteigenvalue}$$

First, the scores of the three variables representing education level, occupation, and family income were converted into standard scores and subjected to principal component analysis to derive the value of the characteristic root of the first factor. Second, the component matrix was used to derive the coefficient of the educational level of the primary caregiver β_1_, the coefficient of the occupation of the primary caregiver β_2_, and the coefficient of the family income β_3_, on the basis of which the data were entered into the formula for the overall calculation of the scores as the socioeconomic status of the university students’ families, and the higher the score, the higher the socioeconomic status of the family. In this study, Cronbach’s α coefficient is 0.915.

The validity of the questionnaire used in this study was verified using KMO and Bartlett’s test, the coefficient result of the KMO test was 0.977 and the Chi-square value of Bartlett’s test was 21087.018 (Sig. = 0.000 < 0.01).

Table 1 presents the details of factor extraction and the information content of the extracted factors. As can be discerned from the table, the factor analysis extracted four factors, all with eigenvalues >1. After rotation, the variance explained by these four factors are 23.287, 20.018, 17.738, and 7.705%, respectively. The cumulative variance explained post-rotation is 68.749%.

**Table 1:** Variance explained.

Factor ID	Eigenvalue			Variance explained before rotation			Variance explained after rotation		
	Total	Variance explained (%)	Cumulative (%)	Total	Variance explained (%)	Cumulative (%)	Total	Variance explained (%)	Cumulative (%)
1	19.464	39.721	39.721	19.464	39.721	39.721	11.411	23.287	23.287
2	6.628	13.527	53.249	6.628	13.527	53.249	9.809	20.018	43.305
3	4.477	9.137	62.386	4.477	9.137	62.386	8.692	17.738	61.044
4	3.118	6.364	68.749	3.118	6.364	68.749	3.776	7.705	68.749

Extraction method: principal component analysis.

This study employed the Varimax rotation method to rotate the data, determining the relationship between the factors and the research items. Table 2 showcases the information extraction for each factor related to the research items and the corresponding relationships between them. As can be observed from the table, the communalities for all research items exceed 0.4, indicating a robust association between the research items and the factors, suggesting that the factors effectively extract information. While ensuring that the factors capture a majority of the information from the research items, the emphasis of subsequent analyses lies in discerning the specific relationships between the factors and the research items (a factor loading with an absolute value >0.4 signifies a correspondence between the item and the factor).

**Table 2:** Rotated factor loadings.

Item code	Factor loading coefficient				Communality
	Factor 1	Factor 2	Factor 3	Factor 4	
Anxiety and depression (AAD)1	0.126	***0.820***	−0.168	−0.098	0.725
AAD2	0.237	***0.751***	−0.189	−0.144	0.676
AAD3	0.242	***0.776***	−0.208	−0.119	0.719
AAD4	0.240	***0.786***	−0.196	−0.080	0.720
AAD5	0.212	***0.807***	−0.217	−0.101	0.753
AAD6	0.224	***0.790***	−0.178	−0.126	0.722
AAD7	0.209	***0.782***	−0.181	−0.060	0.692
AAD8	0.234	***0.793***	−0.195	−0.078	0.728
AAD9	0.235	***0.809***	−0.180	−0.064	0.746
AAD10	0.259	***0.784***	−0.208	−0.051	0.728
AAD11	0.264	***0.767***	−0.204	−0.112	0.713
AAD12	0.203	***0.778***	−0.236	−0.063	0.706
AAD13	0.188	***0.796***	−0.220	−0.110	0.730
AAD14	0.222	***0.779***	−0.224	−0.099	0.716
Perceived social support (PSS)1	−0.108	−0.184	***0.791***	0.130	0.687
PSS2	−0.095	−0.199	***0.787***	0.066	0.671
PSS3	−0.136	−0.180	***0.779***	0.049	0.660
PSS4	−0.144	−0.187	***0.788***	0.061	0.681
PSS5	−0.160	−0.176	***0.747***	0.076	0.621
PSS6	−0.114	−0.169	***0.761***	0.049	0.623
PSS7	−0.126	−0.175	***0.782***	0.094	0.668
PSS8	−0.128	−0.128	***0.766***	0.090	0.628
PSS9	−0.121	−0.155	***0.789***	0.123	0.676
PSS10	−0.078	−0.221	***0.769***	0.085	0.654
PSS11	−0.104	−0.181	***0.770***	0.034	0.638
PSS12	−0.174	−0.194	***0.769***	0.059	0.663
PSS13	−0.165	−0.231	***0.757***	0.121	0.668
Perceived loss of control (PLOC)1	***0.795***	0.204	−0.108	−0.021	0.686
PLOC2	***0.789***	0.160	−0.132	−0.038	0.667
PLOC3	***0.807***	0.167	−0.157	−0.062	0.707
PLOC4	***0.780***	0.233	−0.134	−0.030	0.681
PLOC5	***0.783***	0.172	−0.136	−0.068	0.666
PLOC6	***0.770***	0.196	−0.181	0.024	0.664
PLOC7	***0.786***	0.200	−0.106	−0.033	0.670
PLOC8	***0.778***	0.192	−0.113	−0.122	0.670
PLOC9	***0.761***	0.156	−0.087	−0.049	0.614
PLOC10	***0.781***	0.164	−0.138	−0.025	0.656
PLOC11	***0.788***	0.169	−0.065	−0.049	0.657
PLOC12	***0.795***	0.208	−0.106	−0.036	0.688
PLOC13	***0.808***	0.161	−0.101	−0.035	0.691
PLOC14	***0.790***	0.168	−0.117	−0.008	0.665
PLOC15	***0.770***	0.210	−0.094	−0.028	0.647
PLOC16	***0.788***	0.175	−0.134	−0.060	0.673
PLOC17	***0.772***	0.167	−0.100	−0.028	0.635
Family SES1	−0.048	−0.172	0.135	***0.849***	0.771
Family SES2	−0.074	−0.089	0.140	***0.824***	0.713
Family SES3	−0.048	−0.165	0.107	***0.861***	0.782
Family SES4	−0.078	−0.173	0.144	***0.832***	0.749
Family SES5	−0.061	−0.138	0.160	***0.821***	0.722

Extraction method: principal component analysis. Rotation method: Kaiser normalization with maximum variance. Numbers in the highlighted in bold indicate factor loadings with an absolute value >0.4.

### 2.3 Data analysis

In order to test the hypotheses of the proposed model, we performed statistical analysis using SPSS 26.0 and PROCESS software, structured as follows. Demographic descriptive analysis was performed on the sample of subjects. The correlations with the scales and data were first analyzed using Cronbach’s coefficient for the reliability test and Harman but for the causal play for the common method bias analysis, and then constructing the correlation of the variables to analyze the correlation and the degree of correlation between the variables. The mediation analysis and moderated effects were conducted in conjunction with Hayes (101) PROCESS model4 and model8. A moderated mediation effect can be considered to be present if the bootstrap confidence interval does not include zero.

## 3 Results

Data variables for four variables, anxiety and depressed, social support, perceived loss of control, and family socioeconomic status, were tested for normality. The absolute values of kurtosis were all less than 3, and the current data distribution flat state approximates normal distribution. The skewness is all around 0, and the current data distribution is shifted to approximate a normal distribution. Correlation analysis of the four variables showed that social support was negatively correlated with anxiety and depression (*r* = −0.488, *p* < 0.01), and negatively correlated with perceived loss of control (*r* = −0.345, *p* < 0.01). Perceived loss of control was positively correlated with anxiety and depressed (*r* = 0.499, *p* < 0.01). As shown in the Table 3.

**Table 3:** Correlation analysis of the four variables.

	Mean	Standard deviation	Anxiety and depression	Social support	Perceived loss of control	Family SES
Anxiety and depression	2.380	0.995	1			
			
Social support	3.915	0.854	−0.488^**^	1		
			0.000			
Perceived loss of control	2.299	0.916	0.499^**^	−0.345^**^	1	
			0.000	0.000		
Family SES	4.049	0.944	−0.322^**^	0.297^**^	−0.177^**^	1
			0.000	0.000	0.000	

^**^Significant correlation at 0.01 level (two-tailed).

**Figure 2:**
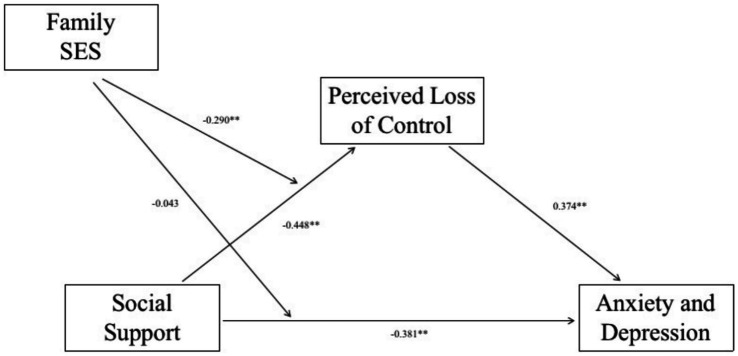
Test results of the mediator model with moderation. **p < 0.01.

The mediation effect analysis was performed using the bootstrap method in the Process macro program, combined with the stepwise test using model4, and the regression analysis showed that social support had a significant negative effect on the perceived loss of control (*β* = −0.37, *t* = −8.223, *p* = 0.000 < 0.01). The perceived loss of control (*β* = 0.404, *t* = 9.591, *p* = 0.000 < 0.01) and social support (*β* = −0.412, *t* = −9.130, *p* = 0.000 < 0.01) significantly influenced anxiety and depression (*R*^2^ = 0.349, *F* = 133.8347, *p* < 0.01). These results suggest that social support negatively affects anxiety and depressive mood and that this relationship is mediated by a sense of loss of control (see Table 4). In addition, mediated effects with moderation were analyzed using Model8 through the bootstrap method in the Process macro program (see Figure 2). Social support (*β* = −0.448, *t* = −9.576, *p* = 0.000 < 0.05) and family socioeconomic status (*β* = −0.177, *t* = −4.159, p = 0.000 < 0.05). Social support*family socioeconomic status (interaction term) on the perceived loss of control (*β* = −0.29, *t* = −7.435, *p* = 0.000 < 0.05), so social support, family socioeconomic status, and the interaction term have a significant negative effect on the sense of loss of control. Social support (*β* = −0.381, *t* = −7.677, *p* = 0.000 < 0.05). Family socioeconomic status (*β* = −0.187, *t* = −4.422, *p* = 0.000 < 0.05). Social support * family socioeconomic status (interaction term) (*β* = −0.043, *t* = −1.059, *p* = 0.290 > 0.05). Perceived loss of control (*β* = 0.374, *t* = 8.554, *p* = 0.000 < 0.05). This shows that social support, family socioeconomic status, has a significant negative effect on anxiety and depression and perceived loss of control has a significant positive effect on anxiety and depression. While the interaction term social support*family socioeconomic status (interaction term) has no effect on anxiety and depression (see Table 4).

**Table 4:** Summary of regression models.

Variables	Perceived loss of control				Anxiety and depression			
	*β*	*t*	LLCI	ULCI	*β*	*t*	LLCI	ULCI
Constant	2.369	63.071	2.295	2.442	1.537	13.992	1.321	1.753
Social support	−0.448	−9.576	−0.540	−0.356	−0.381	−7.677	−0.479	−0.284
Family SES	−0.177	−4.159	−0.260	−0.093	−0.187	−4.422	−0.270	−0.104
Social support*Family SES (interaction term)	−0.290	−7.435	−0.367	−0.213	−0.043	−1.059	−0.121	0.036
Perceived loss of control					0.374	8.554	0.288	0.460
*R* ^2^	0.213				0.374			
*F*	44.8107^**^				74.2595^**^			

^*^p < 0.05, ^**^p < 0.01.

**Figure 3:**
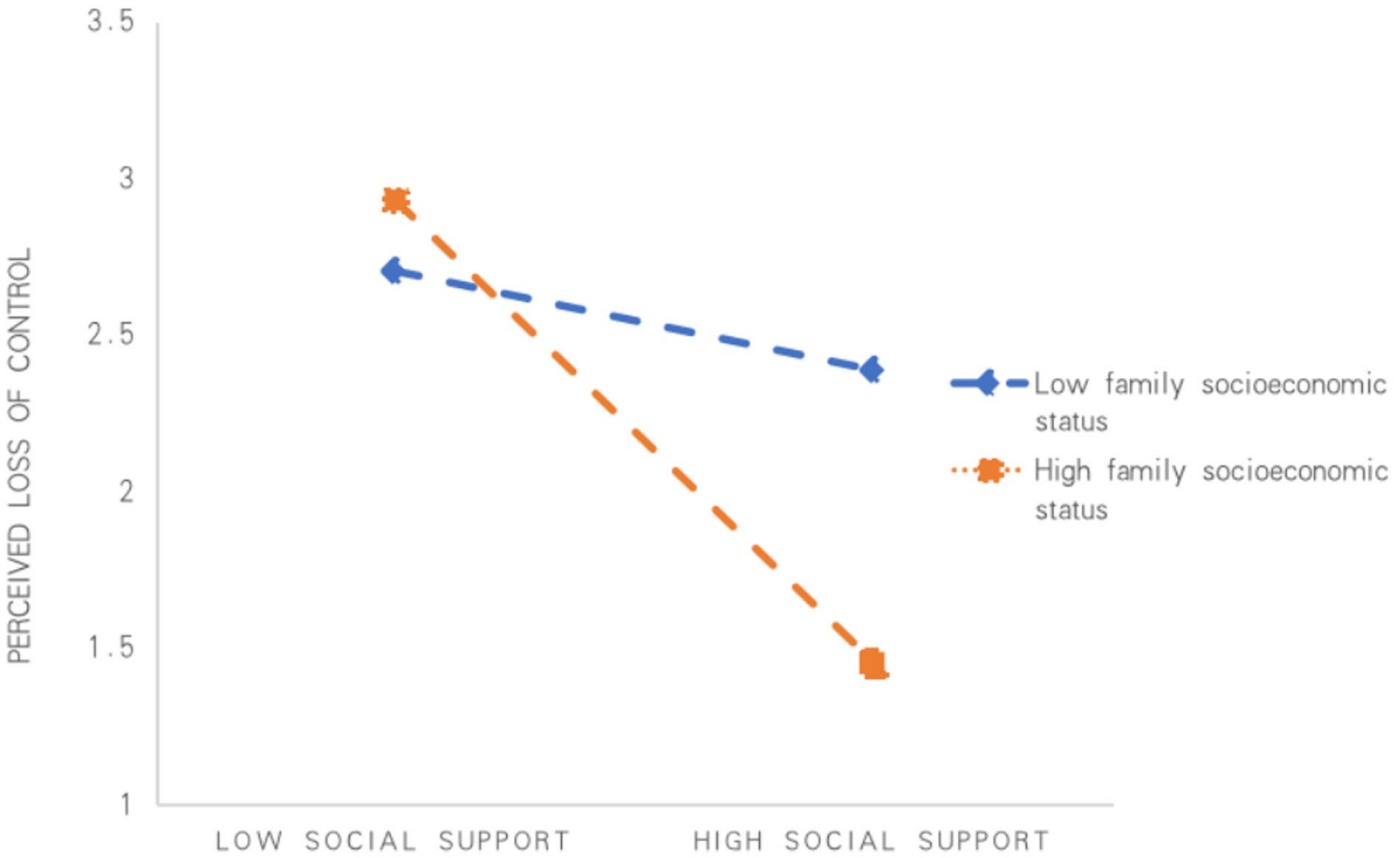
Mediating moderating effect between social support and anxiety and depression.

When analyzing the moderating effect of family socioeconomic status, moderation was found to be significant at (M − 1SD), M, and (M + 1SD), with effect values of −0.341, −0.381, and −0.422, respectively, with 95% CIs of [−0.439, −0.244], [−0.479, −0.284] and [−0.565, −0.278] (see Table 5). Thus, the mediating effect of family socioeconomic status on the effect of social support on the perceived loss of control was significant and varied across the different dimensions, suggesting that the mediation was moderated. A simple slope analysis (see Figure 3) showed that perceived loss of control was significantly reduced as the level of social support increased, and that the higher the family socioeconomic status, the greater the degree of moderation of perceived loss of control. This finding suggests that family socioeconomic status significantly enhances the mediating effect of social support on anxiety and depression.

**Table 5:** Results of the conditional indirect effect.

Level of moderating variable	Effect	*s.e*.	*t*	*p*	LLCI	ULCI
Low level (M − 1SD)	−0.341	0.050	−6.892	0.000	−0.439	−0.244
Mean value	−0.381	0.050	−7.677	0.000	−0.479	−0.284
High level (M + 1SD)	−0.422	0.073	−5.762	0.000	−0.565	−0.278

Because the different dimensions of social support differed in terms of family socioeconomic status, to further explore the role of the dimensions in social support, the mediating effect of perceived loss of control with moderation between the dimensions of social support and anxiety and depressive mood was analyzed using model8 in the Process macro program.

As illustrated in Tables 6–8, family support (*β* = −0.384, *t* = −8.496, *p* = 0.000 < 0.05), friend support (*β* = −0.396, *t* = −8.911, *p* = 0.000 < 0.05), other support (*β* = −0.423, *t* = −9.346, *p* = 0.000 < 0.05), family support*family socioeconomic status (interaction term) (*β* = −0.252, *t* = −6.482, *p* = 0.000 < 0.05), friend support*family socioeconomic status (interaction term) (*β* = −0.264, *t* = −6.908, *p* = 0.000 < 0.05), and other support*family socioeconomic status (interaction term) (*β* = −0.274, *t* = −7.288, *p* = 0.000 < 0.05) all had a significant negative effect on perceived loss of control, but no effect on either anxiety or depression.

**Table 6:** Summary of regression models for dimensions of family support.

Variable	Perceived loss of control				Anxiety and depression			
	*β*	*t*		*β*	*t*		*β*	*t*
Constant	2.358	61.742	2.283	2.433	1.471	13.606	1.259	1.683
Family support	−0.384	−8.469	−0.473	−0.295	−0.333	−7.143	−0.425	−0.242
Family socioeconomic status	−0.173	−4.037	−0.258	−0.089	−0.181	−4.310	−0.263	−0.098
Family support*Family SES (interaction term)	−0.252	−6.482	−0.328	−0.176	−0.005	−0.114	−0.081	0.072
Perceived loss of control					0.399	9.252	0.314	0.484
*R* ^2^	0.181				0.367			
*F*	36.6858^**^				72.0518^**^			

^*^p < 0.05, ^**^p < 0.01.

**Table 7:** Summary of regression models for dimensions of friend support.

Variable	Perceived loss of control				Anxiety and depression			
	β	t		β	t		β	t
Constant	2.362	62.382	2.287	2.436	1.493	13.557	1.276	1.709
Friend support	−0.396	−8.911	−0.484	−0.309	−0.315	−6.711	−0.407	−0.223
Family SES	−0.182	−4.254	−0.267	−0.098	−0.200	−4.679	−0.284	−0.116
Friend support*Family SES (interaction term)	−0.264	−6.908	−0.339	−0.189	−0.036	−0.919	−0.113	0.041
Perceived loss of control					0.393	8.949	0.307	0.479
*R* ^2^	0.196				0.356			
*F*	40.3588^**^				68.6067^**^			

^*^p < 0.05, ^**^p < 0.01.

**Table 8:** Summary of regression models for dimensions of other support.

Variable	Perceived loss of control				Anxiety and depression			
	*β*	*t*		*β*	*t*		*β*	*t*
Constant	2.367	62.796	2.293	2.441	1.533	14.035	1.319	1.748
Other support	−0.423	−9.346	−0.512	−0.334	−0.370	−7.761	−0.463	−0.276
Family SES	−0.178	−4.168	−0.262	−0.094	−0.187	−4.429	−0.269	−0.104
Other support*Family SES (interaction term)	−0.274	−7.288	−0.348	−0.200	−0.041	−1.072	−0.117	0.034
Perceived loss of control					0.376	8.639	0.290	0.461
*R* ^2^	0.206				0.376			
*F*	43.0307^**^				74.7078^**^			

^*^p < 0.05, ^**^p < 0.01.

As can be seen in Tables 9–11, under family, friends, and other support, both families with lower family socioeconomic status (M-1SD) and families with higher family socioeconomic status (M + 1SD) had significant negative effects on perceived loss of control. And under different dimensions of social support, family support in higher family socioeconomic status (*β* = −0.338, *t* = −4.933, *p* < 0.05) had an effect value of −0.338, with a 95% CI not containing 0. Friend support (*β* = −0.349, *t* = −5.054, *p* < 0.05) had an effect value of −0.349, with a 95% CI not including 0. Other support (*β* = −0.408, *t* = −5.835, *p* < 0.05) had an effect value of −0.408, with a 95% CI not including 0. All these findings indicate a stronger mitigating effect on college students’ perceived loss of control during home isolation.

**Table 9:** Results of conditional indirect effects on dimensions of family support.

Level of moderating variables	Effect	*s.e.*	*t*	*p*	LLCI	ULCI
Low level (M − 1SD)	−0.329	0.049	−6.757	0.000	−0.425	−0.233
Mean value	−0.333	0.047	−7.143	0.000	−0.425	−0.242
High level (M + 1SD)	−0.338	0.068	−4.933	0.000	−0.472	−0.203

**Table 10:** Results of conditional indirect effects on dimensions of friend support.

Level of moderating variables	Effect	*s.e.*	*t*	*p*	LLCI	ULCI
Low level (M − 1SD)	−0.281	0.049	−5.760	0.000	−0.377	−0.185
Mean value	−0.315	0.047	−6.711	0.000	−0.407	−0.223
High level (M + 1SD)	−0.349	0.069	−5.054	0.000	−0.484	−0.213

**Table 11:** Results of conditional indirect effects on dimensions of other support.

Level of moderating variables	Effect	*s.e*.	*t*	*p*	LLCI	ULCI
Low level (M − 1SD)	−0.331	0.048	−6.955	0.000	−0.424	−0.237
Mean value	−0.370	0.048	−7.761	0.000	−0.463	−0.276
High level (M + 1SD)	−0.408	0.070	−5.835	0.000	−0.546	−0.271

## 4 Discussion

Empirical studies suggest that, influenced by a family’s socioeconomic status, the enhancement of various social support ecosystems (including family, friends, and other forms of support) more markedly reduces the perceived loss of control among families with a higher socioeconomic status compared to those with a lower one. This underscores the pivotal moderating role that family socioeconomic status plays in the relationship between social support and perceived loss of control. Compared to other studies that have found the impact of support from family and friends on an individual’s emotions during pandemics, the assistance, particularly from the community, streets, and neighborhoods, plays an indispensable role in alleviating the perceived loss of control among quarantined college students.

Furthermore, in the post-pandemic era, social support has exerted a protective buffering effect on college students’ emotions, primarily mediated by the perceived loss of control. Family socioeconomic status further modulates this mediating effect. The interaction between social support and anxiety and depression (with perceived loss of control as the mediator) indicates that social support can significantly alleviate both anxiety and depression, thereby confirming *Hypothesis 1*. Through its influence on perceived loss of control, social support indirectly mitigates anxiety and depression, validating *Hypothesis 2*. As the degree of social support increases, the perceived loss of control among college students noticeably diminishes, leading to a reduction in anxiety and depression. The effect of social support on the perceived loss of control depends on family socioeconomic status. The mediating role of perceived loss of control in the relationship between social support and anxiety and depression becomes more pronounced across different socioeconomic backgrounds, further endorsing *Hypothesis 3*.

Research indicates that while college students indeed experience anxiety and depression during periods of isolation from their families, these negative emotions are influenced by factors such as social support, a perceived loss of control, and family socioeconomic status. Social support is a multifaceted system, encompassing both emotional and material backing from family and friends, as well as information and tangible assistance from the broader community (102–104). In environments of seclusion, care from relatives, material aid from communities, and information exchanges among peers all contribute to cultivating a sense of belonging and compassion in college students, alleviating their feelings of anxiety and depression (105). Notably, support from friends holds particular significance. This is likely because the Chinese government’s home isolation policy coincided with the academic term, amplifying the importance of this support due to the unique situation of college students.

In the post-pandemic period, especially during home quarantine, community support acts as the foundation for all supportive mechanisms, especially within the framework of the home isolation policy (106). Local communities and neighborhoods proactively care for each resident’s welfare, distribute essential items, and provide indispensable social backing (107, 108). Alongside tangible support, there is a heightened focus on mental well-being. Studies have shown that reducing the perceived loss of control in college students can significantly lessen their anxiety and depression, subsequently influencing their outlook on future academic and personal pursuits. When individuals feel they are losing control in their personal and professional lives, it can lead to a pessimistic view of the future, potentially triggering panic. Such feelings can have lasting effects on their daily routines and long-term goals.

The relationship between social support and feelings of anxiety and depression is largely influenced by family socioeconomic status and the sense of losing control. Specifically, as family socioeconomic status rises, social support becomes more effective in alleviating negative emotions by suppressing the perceived loss of control. Family socioeconomic status not only reflects an individual’s perceived resources and societal standing but also profoundly impacts a college student’s quality of life, emotional perception, and coping mechanisms. Generally, individuals from higher socioeconomic backgrounds may exhibit stronger logical reasoning and decision-making capabilities (109, 110). Conversely, compared to students from affluent families, those from families with lower educational and income levels might face restrictions in accessing materials and resources (111, 112), potentially heightening their risk of anxiety and depression during pandemics.

Students from higher socioeconomic backgrounds display distinct advantages in information access, emotional regulation, and resource acquisition, which in turn reduces anxiety and depression during home isolation. In contrast, students from lower socioeconomic standings exhibit elevated levels of anxiety and depression, with concerns about their academic and personal futures possibly exacerbating these feelings. Given their typically limited family reserves and the pandemic’s impact on their education, these students might grapple with escalating anxiety and depression as isolation persists. Hence, prioritizing their mental well-being is essential. We advocate for educational institutions and community organizations to provide enhanced psychological support to these students, guiding them to view the pandemic from a balanced perspective and alleviate their distress.

In this study, we explored the impact of social support on the emotions of college students, particularly during home isolation in the post-pandemic era. Research indicates that support from friends, encompassing emotional comfort, encouragement, and practical information exchange, positively influenced college students. Furthermore, the wider community, serving as an extended social entity, made significant contributions by providing housing and medical assistance. This external support alleviated the students’ perceived loss of control, an emotion that intensifies during isolation and major pandemics. From a psychological perspective, a perceived loss of control is inherently linked to anxiety and depression. When individuals feel they lack control over their lives and futures, it gives rise to feelings of anxiety, concern, and even panic. However, such emotions tend to diminish with external support and understanding. Hence, our research emphasizes the profound role of multifaceted social support in alleviating anxiety and depression among college students. This insight offers a novel perspective, highlighting the potential of bolstering social support to enhance individual mental health within specific socio-cultural contexts. Such understanding not only furnishes actionable insights for mental health professionals but also guides policymakers in formulating effective support mechanisms during public health crises.

### 4.1 Main contributions

This study elucidates several novel theoretical viewpoints. Firstly, it was discovered that college students’ perceived loss of control serves as a partial mediator between social support and feelings of anxiety and depression. Additionally, family socioeconomic status moderates this mediating effect, highlighting the intricate dynamic relationship between social support and emotional distress in college students. Secondly, through an analysis of family socioeconomic status, the study offers fresh insights into research related to college students’ anxiety and depression in the post-pandemic era. Lastly, this investigation deepens our understanding of the potential mechanisms influencing negative emotions, offering suggestions for more targeted intervention measures.

### 4.2 Practical implications

This article, based on empirical research, proposes the following recommendations to enhance social support and alleviate the emotional distress of college students isolated at home during the pandemic:

Disseminate accurate pandemic information: College students’ anxiety and perceived loss of control largely stem from their limited understanding of the pandemic and concerns about the future. Providing them with accurate information promptly can foster a positive mindset, thereby reducing psychological stress.

Address specific psychological needs: During home isolation, some students may experience anxiety due to academic challenges, health concerns, or job-seeking pressures. Communities and educational institutions must recognize and cater to these students’ needs, offering targeted psychological support and resource sharing.

Enhance the provision of online resources: Educational institutions should amplify the dissemination of online academic and employment-related resources. This can assist students in better planning for their future, consequently reducing feelings of anxiety and depression.

Adopt a holistic intervention strategy: Our research emphasizes the pivotal role of support from family, friends, and other social networks in mitigating college students’ perceived loss of control and associated negative emotions. Notably, assistance from communities and neighborhoods during the post-pandemic period plays an indispensable role in regulating students’ emotions. Moreover, a family’s socioeconomic status significantly moderates the students’ perceived loss of control. We advocate for an integrated intervention approach that consolidates various resources to bolster the mental health of college students during the pandemic.

By implementing these strategies, we can not only alleviate the negative emotions of college students during the pandemic but also foster a more conducive environment for their holistic development.

### 4.3 Limitations

During home isolation, college students face confinement, significantly limiting their interactions with the external environment and impeding their ability to access information promptly. Concurrently, concerns about potential infections and dwindling resources intensify negative emotions. Undeniably, this scenario heightens psychological stress among college students, potentially triggering a range of mental health issues. This study empirically examined the interplay between social support, perceived loss of control, family socioeconomic status, and the resultant feelings of anxiety and depression, aiming to identify strategies to alleviate psychological stress during isolation. However, this research primarily centers on a specific group of college students, excluding a broader population and lacking cross-regional comparisons or evaluations under varying pandemic intensities. Subsequent studies might delve deeper into these aspects, exploring other determinants and mechanisms influencing negative emotions during pandemics, beyond the impacts of perceived loss of control and family socioeconomic status. These considerations pave the way for future explorations. Lastly, while this study amassed extensive cross-sectional data, in discussing potential risks in the post-pandemic era, it provides only correlational rather than causal evidence. Hence, this study cannot fully elucidate the psychological shifts among college students. Future research might employ longitudinal studies to probe into mental health issues in the post-pandemic age.

## 5 Conclusion

This study indicates that during the post-pandemic period, there is a negative correlation between social support and anxiety and depression among college students in home isolation. In this relationship, the perceived loss of control acts as a partial moderating factor, while family socioeconomic status is believed to influence this moderating effect. Notably, among college students with higher family socioeconomic status, the modulating effect of social support on anxiety and depression (with perceived loss of control as a mediator) is more pronounced, and the support from community neighborhoods and the like played a significant role during this pandemic. These findings offer a deeper understanding of the role of social support in alleviating negative emotions such as anxiety and depression in the post-pandemic era. They provide a foundation for formulating more effective intervention strategies during crises, thereby mitigating negative emotions and promoting mental well-being.

## Data availability statement

The original contributions presented in the study are included in the article/supplementary material, further inquiries can be directed to the corresponding author.

## Ethics statement

The studies involving humans were approved by the Institutional Review Board of Shanghai Jiao Tong University. The studies were conducted in accordance with the local legislation and institutional requirements. The participants provided their written informed consent to participate in this study. Written informed consent was obtained from the individual(s) for the publication of any potentially identifiable images or data included in this article.

## Author contributions

HS: Writing – original draft.

## Footnotes

^1^ http://www.sojump.com
